# Clinical observation of posterior scleral reinforcement in the treatment of progressive high myopia in Chinese children: a retrospective study

**DOI:** 10.1186/s12886-022-02375-1

**Published:** 2022-04-01

**Authors:** Shouming Gao, Yuanlong Liu, Shuting Ma, Lixia Li, Yanyan Mao

**Affiliations:** Fuzhou Children’s Hospital of Fujian Medical University, Fuzhou, China

**Keywords:** High myopia in children, Axial length, Corneal radius of curvature, Posterior scleral reinforcement surgery

## Abstract

**Objective:**

To observe the efficacy and safety of posterior sclera reinforcement over time.

**Methods:**

This retrospective single-arm cohort study included children with high myopia who underwent the modified Snyder-Thompson type posterior sclera reinforcement surgery from 03/2015 to 08/2018 at Fuzhou Children’s Hospital of Fujian Medical University. Axial length (AL), corneal radius of curvature (CRC), AL/CRC, refractive error, and best-corrected visual acuity (BCVA) were observed from 1 year before the operation to 2 years after.

**Results:**

Nineteen children (33 eyes) with high myopia were included. The patients were 4.9 ± 2.7 (range, 2–10) years of age (three patients were 10 years old, all others were ≤ 7 years old). AL increased from 1 year before surgery to 2 years after surgery (from 25.31 ± 1.59 to 26.76 ± 1.52, *P* < 0.001). The refractive error was smaller 1 year before surgery than at the other timepoints (all *P* < 0.05). BCVA improved over time (*P* < 0.001). Changes over time were also observed in horizontal CRC (hCRC), AL/hCRC, AL/vertical CRC (vCRC), and AL/CRC (all *P* < 0.001), but not in vCRC (*P* = 0.304). The increase of AL at 2 years after surgery was smaller than at 1 year before surgery and 1 year after surgery (both *P* < 0.001). The increase of AL/CRC at 2 years after surgery was smaller than at 1 year before surgery (0.04 ± 0.04 vs. 0.07 ± 0.04; *P* = 0.008).

**Conclusion:**

In the short term, posterior scleral reinforcement surgery can delay the increase of AL of progressive high myopia.

**Supplementary Information:**

The online version contains supplementary material available at 10.1186/s12886-022-02375-1.

## Background

Myopia, especially high myopia, is one of the leading causes of harm to public visual health, and its global prevalence is increasing [1, 2]. There are many high school students with myopia in North America, East Asia, and Europe [1–4]. An alarming feature of the epidemiology of myopia is the increase in the prevalence of high myopia among young people [1, 2]. In urban areas of East Asia, the prevalence of high myopia in school-age children is higher than in adults [3, 5–8]. This situation is problematic: with the gradual increase in axial length (AL), high myopia can cause many ocular complications such as retinal detachment, choroid neovascularization, and macular hemorrhage [9, 10]. Therefore, preventing and controlling the progression of myopia is a critical public health issue.

Progressive high myopia [11] includes continuous axial growth, progressive thinning of the sclera, and localized posterior scleral staphyloma [12, 13]. The axial length has the highest correlation with the number of refraction under the condition of constant corneal refraction [14]. Therefore, the control of axial overgrowth during child development might be crucial for maintaining normal visual development in children and might be a major goal for preventing pathological myopia complications in the future.

Posterior sclera reinforcement was initially proposed by Shevelev and later improved and simplified by Thompson [15]. Posterior sclera reinforcement aims to control AL growth and prevent high myopia complications by strengthening the posterior sclera with grafts. Early studies suggested the safety and effectiveness of this operation [16–18], but with a low level of evidence. With the increase in AL, the cornea may become flatter, but some data suggest that the cornea may also become steeper [19]. Considering that the type of refractive error of the eye is related to the AL and other refractive components (e.g., cornea and lens) [20], it is not sufficient to simply focus on the changes in AL. The AL/corneal radius of curvature (AL/CRC) is related to myopia progression [21–24]. An AL/CRC value is greater than 3 indicates the progression of myopia.

Nevertheless, Curtin & Whitmore [25] reported unsatisfactory results of posterior scleral reinforcement because it did not produce enough convincing evidence of effectiveness. Therefore, this study aimed to observe the efficacy and safety of posterior sclera reinforcement over time. The data could provide additional insight into the measures associated with myopia.

## Methods

### Study design and patients

This retrospective single-arm cohort study included children with high myopia who underwent the modified Snyder-Thompson posterior sclera reinforcement surgery from March 2015 to August 2018 at the Ophthalmology Department of Fuzhou Children’s Hospital of Fujian Medical University. This study was approved by the Ethics Committee of Fuzhou Children’s Hospital of Fujian Medical University (2019–14). The requirement for individual consent was waived by the committee because of the retrospective nature of the study. The study was carried out in accordance with the applicable guidelines and regulations.

The inclusion criteria were 1) progressive high myopia defined as spherical equivalent refraction (SE) ≥ 4.0 diopters (D) [11] after mydriasis and optometry, with an annual increase of SE ≥ 1.0 D, 2) AL > 23.5 mm, and 3) ≤ 10 years of age. The exclusion criteria were 1) corneal and lens-derived non-axial myopia, 2) eye diseases that can affect visual function (e.g., nystagmus, glaucoma, cataract, retinal detachment, eye trauma, macular or peripheral retinopathy, etc.), 3) systemic diseases that can affect the examination results, or 4) history of other eye surgeries (e.g., refractive surgery, scleral buckling, vitrectomy, etc.).

Myopia can be divided into two types: simple myopia and pathological myopia [26]. Patients with simple myopia can achieve a good vision through proper optical correction. In contrast, patients with pathological myopia cannot achieve good eyesight after myopia correction, the myopia degree deepens quickly, and the eye is prone to fundus lesions. Pathological myopia is often diagnosed when myopic fundus retinopathy is diagnosed, but such lesions are rare in children. Therefore, the children were observed for 1 year before surgery to be sure that they had pathological myopia characterized by a fast progression. Of course, posterior scleral reinforcement surgery is not indicated for simple myopia, and it would be unethical to perform such surgery in children in whom simple spectacles can achieve a proper vision. In addition, the study center is a tertiary ophthalmology center, and only the complex cases are referred there. Therefore, the control group would not be representative of the children with high myopia.

### Surgical methods

**Fig. 1:**
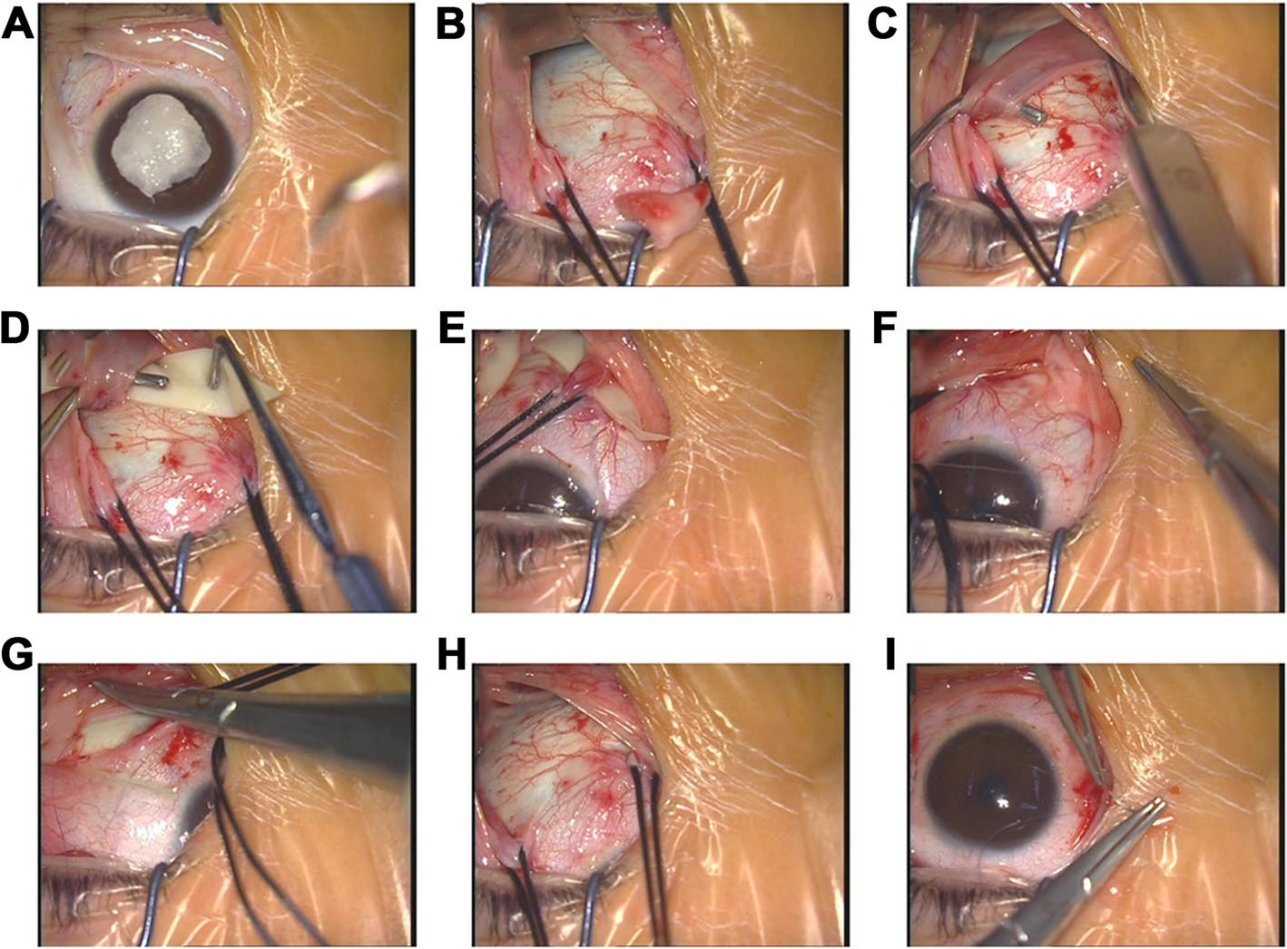
The steps of posterior scleral reinforcement. **A** After eyelid opening, the conjunctiva was cut from the nasal side of the inferior rectus muscle to the conjunctiva of the superior temporal side of the external rectus muscle, at a distance of about 2–3 mm from the limbus of the cornea. **B** The fascia was separated, the inferior and external rectus muscles were exposed, and a traction line was placed and applied upward to the nose. **C** After the macular retractor exposed the inferior oblique muscle, the oblique hook was used to hook out the inferior oblique muscle completely, and the end of the inferior oblique muscle was fully separated. **D** A pericardial patch was placed under the inferior oblique muscle. **E** A pericardial patch was placed under the inferior rectus muscle. **F** After the pericardial patch was straightened to the back of the eyeball, the patch was observed and confirmed to be close to the sclera without wrinkles or distortion. The inferior rectus muscle was sutured nasally and fixed on the sclera, then sutured and fixed with one stitch. **G** The pericardial patch was passed under the external rectus muscle. After the patch was straightened, the patch was observed and confirmed to be close to the sclera without wrinkles or distortion. Suprascleral suture and fixation of the external rectus muscle were performed using one stitch. **H** The eyeball was pulled to the top of the nose, and the lower oblique muscle was exposed using the macular retractor. The patch was seen to be laid flat on the projection position of the macular area above the posterior sclera. The patch was observed and confirmed to be close to the sclera without wrinkles or distortion. **I** After removal of the traction line, the conjunctival incision was sutured in counterposition

All surgeries were performed by the same surgeon with 10 years of experience and under an ophthalmic surgical microscope. Bovine pericardial patch strips with a 6–8-mm width and a length of about 45 mm soaked in anhydrous ethanol were taken out and placed in 500 ml of sterile physiological saline. The bovine pericardial patch used in this study was from Beijing Balance Medical Science and Technology Co. (Beijing, China) and approved by the China Food and Drug Administration. After endotracheal intubation, general anesthesia and routine disinfection and draping were performed. After the eyelid was opened using an eyelid opener, a fan-shaped incision of about 120° was made at 2–3 mm away from the corneal limbal to open the bulbous conjunctiva with the inferior temporal bulbous conjunctiva as the center (Fig. 1A). The subconjunctival fascia was bluntly separated to expose the sclera (Fig. 1B). Traction lines were placed under the lateral rectus and inferior rectus muscles. The external rectus and inferior rectus muscles were pulled in the direction above the nose. The inferior temporal bulbar conjunctiva and fascia were pulled open with a macular hook to completely hook off the inferior oblique muscle and expose the inferior oblique muscle’s end (Fig. 1C). The pericardial patch strips were successively passed through the inferior rectus, inferior oblique, and lateral rectus muscles (Fig. 1D). The strips were laid flat on the macular area’s projection position on the posterior sclera (Fig. 1E). The two ends of the strips were fixed vertically on the superior temporal and the inferior nasal sclera (Fig. 1F). The strips were observed and confirmed to be closely attached to the sclera, with no fold or distortion, no compression of the vortex vein or optic nerve. The strips were located between the inferior oblique muscle and the optic nerve (Fig. 1G, H). The conjunctiva was sutured continuously (Fig. 1I), tobramycin dexamethasone eye cream was applied, and pressure bandaging was performed. After surgery, 0.5% levofloxacin eye drops and 0.1% fluorometholone eye drops were used for 3 weeks to prevent infection and postoperative reaction.

### Outcomes

All children underwent comprehensive ophthalmological examinations during the first visit, preoperative and regular postoperative review, including BCVA, intraocular pressure (rebound tonometer, iCare Finland Oy, Vantaa, Finland), and AL, CRC, refractive error, and slit-lamp examination (Chongqing Kanghua, China) of the anterior segment. AL, horizontal CRC (hCRC), vertical CRC (vCRC), and average CRC were measured using the Lenstar 900 biometric instrument (HAAG-STREIT Diagnostics AG, Koeniz, Switzerland). The average CRC was half of the sum of the hCRC and vCRC. The data was obtained by taking the average value after five consecutive measurements. RD was measured using a computerized automatic optometry instrument (RM-8900, Topcon, Tokyo, Japan) with 0.5% tropicamide eye drops applied once every 5 min, consecutively for five times, and then 30 min later. The refractive error (RD) was recorded by the SE data on the optometry list. The SE value was obtained by summing the value of the spherical degree and half of the value of the astigmatism degree. The data was obtained by taking the average value after five consecutive measurements. Using the E-word Snellen visual acuity chart, BCVA was defined as the smallest straight line with three out of five letters correctly read. The BCVA was converted to LogMAR for statistical analysis.

The same optometrist with a senior professional title performed all examinations. The patients were evaluated 1 year before surgery, at the time of surgery, 1 year after surgery, and 2 years after surgery. A comprehensive ophthalmologic examination was performed at each evaluation, including intraocular pressure, AL, SE, BCVA, and CRC.

### Data collection

The patient’s baseline condition and ocular assessment data at 1 year before surgery, preoperative examination at the time of surgery, 1 year after surgery, and 2 years after surgery were collected from the clinical medical records.

### Statistical analysis

SPSS 26.0 (IBM, Armonk, NY, USA) was used for statistical analysis. The continuous data were presented as means ± standard deviations and analyzed using univariable repeated measures analysis of variance, with the Bonferroni post hoc test. Categorical data were presented as n (%). *P*-values < 0.05 were considered statistically significant.

## Results

### Characteristics of the patients

Nineteen children (33 eyes) with high myopia were included. There were six males and 13 females. The patients were 4.9 ± 2.7 (range, 2–10) years of age. All patients were observed for 1 year before surgery and were followed at 1 and 2 years after surgery. The grafts were in place during the postoperative follow-up period, without infection, severe rejection, or peripheral retinopathy. The observational indexes before surgery (sex, age, AL, SE, BCVA, hCRC, vCRC, AL/hCRC, AL/vCRC, and AL/CRC) are shown in Table 1.

**Table 1:** Characteristics of the patients

Characteristics	*N* = 19
Male/female	6 /13
Age, years (mean ± SD)	4.9 ± 2.7
Affected eyes, n (%)	
Unilateral	5 (26.3)
Bilateral	14 (73.7)
AL, mm (mean ± SD)	25.92 ± 1.55
Refractive error, D (mean ± SD)	-10.54 ± 3.20
BCVA, logMAR (mean ± SD)	0.39 ± 0.25
hCRC, mm (mean ± SD)	7.81 ± 0.24
vCRC, mm (mean ± SD)	7.48 ± 0.26
AL/hCRC (mean ± SD)	3.32 ± 0.18
AL/vCRC (mean ± SD)	3.39 ± 0.222
Al/CRC (mean ± SD)	3.39 ± 0.19

*AL* axial length, *BCVA* best-corrected visual acuity, *CRC* corneal radius of curvature, *hCRC* horizontal corneal radius of curvature, *vCRC* vertical corneal radius of curvature

### Condition of the eyes at different timepoints

Table 2 presents the comparison of the patients’ eye conditions at different timepoints. AL increased progressively from 1 year before surgery to 2 years after surgery (from 25.31 ± 1.59 to 26.76 ± 1.52, *P* < 0.001). The refractive error was smaller 1 year before surgery than at the other timepoints (all *P* < 0.05). BCVA improved over time (*P* < 0.001). Changes over time were also observed in hCRC, AL/hCRC, AL/vCRC, and AL/CRC (all *P* < 0.001), but not in vCRC (*P* = 0.304).

**Table 2:** Comparison of the patients’ eye conditions at different time points

Characteristics (mean ± SD)	12 months before surgery	Baseline	1 year after surgery	2 years after surgery	*P*
AL (mm)	25.31 ± 1.59^abc^	25.92 ± 1.55^de^	26.43 ± 1.48^f^	26.76 ± 1.52	< 0.001
Refractive error (D)	-8.39 ± 2.99^abc^	-10.54 ± 3.20	-10.86 ± 3.16	-10.97 ± 3.14	< 0.001
BCVA (logMAR)	0.44 ± 0.26^bc^	0.39 ± 0.25^de^	0.31 ± 0.22^f^	0.24 ± 0.19	< 0.001
hCRC (mm)	7.78 ± 0.24^abc^	7.81 ± 0.24^e^	7.84 ± 0.24	7.85 ± 0.24	< 0.001
vCRC (mm)	7.46 ± 0.24	7.48 ± 0.26	7.48 ± 0.25	7.48 ± 0.28	0.304
AL/hCRC	3.25 ± 0.18^abc^	3.32 ± 0.18^de^	3.37 ± 0.17^f^	3.41 ± 0.17	< 0.001
AL/vCRC	3.40 ± 0.21^bc^	3.39 ± 0.22^de^	3.54 ± 0.18^f^	3.58 ± 0.20	< 0.001
Al/CRC	3.32 ± 0.19^abc^	3.39 ± 0.19^de^	3.45 ± 0.17^f^	3.49 ± 0.18	< 0.001

*AL* axial length, *BCVA* best-corrected visual acuity, *CRC* corneal radius of curvature, *hCRC* horizontal corneal radius of curvature, *vCRC* vertical corneal radius of curvature

^a^*P* < 0.05, 12 months before surgery vs. Baseline

^b^*P* < 0.05, 12 months before surgery vs. 12 months after surgery

^c^*P* < 0.05, 12 months before surgery vs. 2 years after surgery

^d^*P* < 0.05, Baseline vs. 12 months after surgery

^e^*P* < 0.05, Baseline vs. 2 years after surgery

^f^*P* < 0.05, 12 months after surgery vs. 2 years after surgery

The children were followed for 2 years after surgery. No graft rejection or limited eye movement was observed. Additional file 1 presents the outcomes for all children.

### Changes in eye conditions at different timepoints

**Fig. 2:**
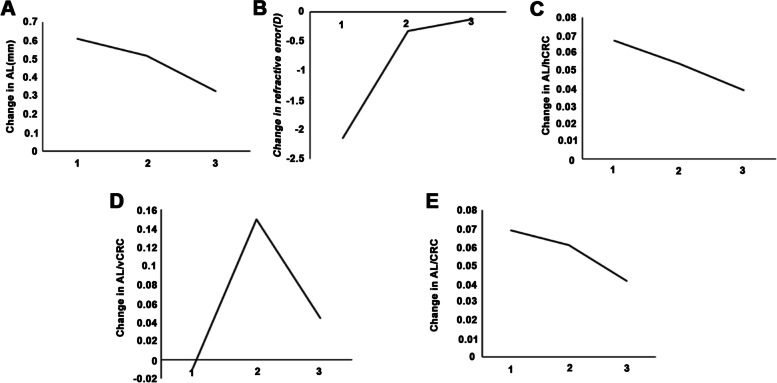
The change of patients’ eye conditions between different time points. **A** The change of Axial length (AL); **B** The change of refractive error; **C** The change of AL/horizontal corneal radius of curvature (AL/hCRC); **D** The change of AL/vertical corneal radius of curvature (AL/vCRC): **E** The change of AL/CRC. 1: △ (baseline-12 months before surgery); 2: △ (1 year after surgery-baseline); 3: △ (2 years after surgery-1 year after surgery)

The increase in AL in the second year after surgery was significantly smaller than during the year before surgery (0.33 ± 0.17 vs. 0.61 ± 0.31 mm; *P* < 0.001) and the first year after surgery (0.33 ± 0.17 vs. 0.52 ± 0.27 mm; *P* < 0.001). The changes in refractive error during the year before surgery were significantly different than during the first (-2.14 ± 0.73 vs. -0.32 ± 0.83 D; *P* < 0.001) and the second (-2.14 ± 0.73 vs. -0.12 ± 0.64 D; *P* < 0.001) years after surgery. During the year before surgery, the increase in AL/hCRC was significantly greater than during the second year after surgery (0.07 ± 0.04 vs. 0.04 ± 0.03; *P* = 0.012). The changes in AL/vCRC during the second year after surgery were significantly different than during the year before surgery (0.04 ± 0.05 vs. -0.01 ± 0.02; *P* < 0.001) and the first year after surgery (0.04 ± 0.05 vs. 0.15 ± 0.07; *P* < 0.001). During the first year after surgery, the changes in AL/vCRC were also significantly different than during the year before surgery (0.15 ± 0.07 vs. -0.01 ± 0.02; *P* < 0.001). The increase in AL/CRC during the second year after surgery was significantly smaller than during the year before surgery (0.04 ± 0.04 vs. 0.07 ± 0.04; *P* = 0.008) (Table 3 and Fig. 2).

**Table 3:** Comparison of the change of patients’ eye conditions between different time points

Characteristics (mean ± SD)	△ (baseline-12 months before surgery)	△ (1 year after surgery-baseline)	△ (2 years after surgery—1 year after surgery)	*P*
AL (mm)	0.61 ± 0.31	0.52 ± 0.27	0.33 ± 0.17*^&^	< 0.001
Refractive error (D)	-2.14 ± 0.73	-0.32 ± 0.83^#^	-0.12 ± 0.64^&^	< 0.001
hCRC (mm)	0.03 ± 0.05	0.03 ± 0.06	0.01 ± 0.05	0.157
vCRC (mm)	0.02 ± 0.05	-0.00 ± 0.08	0.00 ± 0.09	0.471
AL/hCRC	0.07 ± 0.04	0.05 ± 0.04	0.04 ± 0.03^&^	0.008
AL/vCRC	-0.01 ± 0.02	0.15 ± 0.07^#^	0.04 ± 0.05*^&^	< 0.001
AL/CRC	0.07 ± 0.04	0.06 ± 0.04	0.04 ± 0.04^&^	0.008

*AL* axial length, *BCVA* best-corrected visual acuity, *CRC* corneal radius of curvature, *hCRC* horizontal corneal radius of curvature, *vCRC* vertical corneal radius of curvature

^#^*P* < 0.05, △ (1 year after surgery-baseline) vs. △ (baseline-12 months before surgery)

^*^P < 0.05, △ (2 years after surgery—1 year after surgery) vs. △ (1 year after surgery-baseline)

^&^*P* < 0.05, △ (2 years after surgery—1 year after surgery) vs. △ (baseline-12 months before surgery)

## Discussion

This study aimed to observe the efficacy and safety of posterior sclera reinforcement over time. The results strongly suggest that posterior scleral reinforcement surgery can effectively delay AL growth of progressive high myopia. The data provide additional insight into the management of progressive high myopia in children.

High myopia is characterized by axial elongation and posterior sclera thinning, which might be due to the weakened biomechanical properties of the sclera [27–29]. The mechanism by which posterior scleral reinforcement controls rapid AL growth is thought to be through the direct mechanical force of the strips placed on the posterior pole of the eye [30, 31] and/or the posterior polar sclera remodeling and hyperplasia secondary to nonspecific inflammation generated between the posterior sclera and the strips. Posterior sclera reinforcement is based on the use of grafts to reinforce the posterior sclera of the eyeball to prevent the progression of scleral staphyloma of the posterior pole of the eye and the elongation of the AL and control the progression of myopia. Materials used in posterior scleral reinforcement include autologous fascia lata, allogeneic sclera, allogeneic dura mater, and fetal umbilical cord [32–34], but these materials are difficult to obtain and preserve and could cause problems such as rejection and infections [32–34]. The US Food & Drug Administration (FDA) approved a bovine pericardial patch as a dural graft material in 1996 [35]. This material is widely used in cardiac surgery, thoracic surgery, neurosurgery, and other clinical fields [36–38]. Artificial bovine pericardial patch, which is being made more widely available, has the advantages of good biocompatibility, easy modification, and sufficient sources; it can be retained for a long time on the surface of the human sclera and can fuse with the surface sclera [32]. An artificial bovine pericardial patch facilitates the growth of new blood vessels and thus improves the blood supply of the posterior polar sclera [32]. During the study period, the bovine pericardial patch was from Beijing Balance Medical Science and Technology Co. and has been approved by the China Food & Drug Administration. A comparative study on materials for posterior scleral reinforcement showed the good safety and biocompatibility of bovine pericardial patch as a substitute material [39].

In this study, during the 2-year of follow-up after the operation, no postoperative complications were found, as supported by the literature [39]. In this study, posterior scleral reinforcement using the bovine pericardial patch as the reinforcement material could effectively slow down the AL growth rate in children with progressive high myopia. It could be seen from the difference value of AL growth year by year that the AL growth tended to decrease after surgery. This trend was also consistent with a year-on-year decrease in the increase of diopter. Indeed, the average AL growth was 0.61 ± 0.31 before surgery, 0.52 ± 0.27 in the first year after surgery, and 0.33 ± 0.17 in the second year. Similarly, such a trend was also observed on the increase of diopters; the increase rate of a diopter during the first year and the second year after surgery was significantly slower than during the year before surgery. These results are supported by previous studies [16, 40, 41]. No severe complications were found during the two-year follow-up after surgery, suggesting that the bovine pericardial patch can replace the sclera as the material for posterior scleral reinforcement in the future. Regarding the changes in eye AL, the preoperative and 1-year postoperative values were actually not significantly different. Still, they were significant in the second year, suggesting that control improved gradually and that some time might be needed for the patch to stretch and exert its effects. Similar results were observed regarding the degree of myopia. There is a time difference between the control of the eye AL growth and the control of the degree of myopia, which also indicates that AL should not be the only parameter considered in such patients.

However, Curtin & Whitmore [25] reported a mean change in refraction from baseline to end of follow-up of 0.77 D in 23 cases (compared to 0.71 D in 20 controls) over 8 years. No overall significant differences were observed between cases and controls in AL elongation. The high variability in patient baseline characteristics (74% of the patients were 8 to 18 years of age) might account for the difference. The patients in the present study were younger, and the effect might be more pronounced. In contrast to age-related visual acuity loss in adults with progressive high myopia [42, 43], BCVA improved in all children with high myopia after post-scleral reinforcement surgery during the follow-up period, which might be due to the ongoing development of the children’s visual acuity rather than the effect of improved visual acuity after surgery. Therefore, the vision was improved, although it is not the main purpose of the surgery, which is to control AL elongation [15].

Choroidal and macular diseases caused by high myopia are rare in children, but their prevalence increases with age and myopia degree [9, 10]. Therefore, posterior scleral reinforcement has many advantages for the control of high myopia in young children. In the past, only the AL and SE were used to evaluate the effectiveness of posterior scleral reinforcement. Grosvenor [21] was one of the first authors to study the relationship between AL/CRC and diopter. AL/CRC can better correlate with diopter changes than simple AL changes [19, 21, 22]. Grosvenor & Goss [44] and Goss & Jackson [45] suggested that if the AL/CRC ratio is above 3, eyeball growth and development are faster, and diopter changes will tend to be myopic [44, 45]. Therefore, AL/CRC might be a significant index for judging the progression of myopia, in which AL/hCRC might have higher sensitivity and specificity [45]. Still, additional studies are necessary to determine the actual clinical value of this marker.

This study has some limitations. First, this study was retrospective and with a relatively small sample size. Additional studies with larger sample sizes are needed to validate the findings. Second, this was a single-center study, and the generalizability of the findings is unknown. Third, intraocular pressure data were incomplete because of the poor cooperation of the children and could not be analyzed. Fourth, the equivalent spherical mirror method was routinely used in the children, which is more convenient for the analysis but does not provide specific astigmatism data. Fifth, most children, if not all, probably suffered from congenital since acquired myopia usually occurs later in life [46]. Finally, the follow-up was limited to 2 years.

## Conclusions

In conclusion, posterior scleral reinforcement surgery can effectively delay the AL growth of progressive high myopia. Other parameters, such as the AL/CRC ratio changes, could be used to represent the progression of myopia and growth of AL after the operation, but further studies with a larger sample size and longer-term follow-up are needed.

## Supplementary Information

**Additional file 1.** The outcomes of the patients 2 years after surgery.

## Data Availability

The datasets supporting the conclusions of this article are included within the article and its additional files.

## Abbreviations

AL: Axial Length
CRC: Corneal Radius of Curvature
BCVA: Best-Corrected Visual Acuity
RD: Refractive Error
FDA: Food & Drug Administration
