# Supplementary material for: Clinical observation of posterior scleral reinforcement in the treatment of progressive high myopia in Chinese children: a retrospective study
Source: BMC Ophthalmol. 2022 Apr 1;22:147. doi: 10.1186/s12886-022-02375-1 (PMC8976327; doi:10.1186/s12886-022-02375-1)
Supplement: Supplementary file 1 — Additional file 1. The outcomes of the patients 2 years after surgery. [file 12886_2022_2375_MOESM1_ESM.docx]

**Supplementary Table S1.** The outcomes of the patients 2 years after surgery.

| No. | Sex | Age (years) | Affected eyes | BCVA (logMAR) | AL (mm) | hCRC (mm) | vCRC (mm) | Al/CRC | RD (D) |
| --- | --- | --- | --- | --- | --- | --- | --- | --- | --- |
| 1 | F | 2 | R | 0.2 | 25.40 | 7.58 | 7.39 | 3.39 | -11.88 |
|  |  |  | L | 0.2 | 25.12 | 7.69 | 7.40 | 3.33 | -10.75 |
| 2 | F | 3 | R | 0.3 | 25.49 | 7.78 | 7.37 | 3.37 | -7.75 |
|  |  |  | L | 0.3 | 26.21 | 7.79 | 7.26 | 3.48 | -10.00 |
| 3 | F | 5 | R | 0.1 | 23.81 | 7.58 | 7.43 | 3.17 | -4.63 |
|  |  |  | L | 0.1 | 24.36 | 7.63 | 7.45 | 3.23 | -5.25 |
| 4 | F | 6 | R | 0.1 | 27.41 | 7.97 | 7.88 | 3.46 | -11.25 |
|  |  |  | L | 0.1 | 28.05 | 7.97 | 7.71 | 3.58 | -13.0 |
| 5 | F | 4 | R | 0.1 | 25.63 | 7.76 | 7.28 | 3.41 | -9.00 |
| 6 | F | 7 | L | 0.1 | 25.89 | 7.66 | 7.00 | 3.53 | -11.50 |
| 7 | F | 2 | R | 0.4 | 25.63 | 7.71 | 7.20 | 3.44 | -10.63 |
| 8 | F | 10 | L | 0.8 | 29.50 | 8.07 | 7.76 | 3.73 | -16.88 |
| 9 | F | 2 | R | 0.2 | 23.84 | 7.88 | 7.31 | 3.14 | -5.88 |
|  |  |  | L | 0.2 | 24.65 | 7.81 | 7.39 | 3.24 | -7.50 |
| 10 | M | 6 | R | 0.2 | 27.49 | 7.68 | 7.25 | 3.68 | -12.75 |
|  |  |  | L | 0.2 | 27.43 | 7.63 | 7.20 | 3.70 | -12.13 |
| 11 | F | 2 | R | 0.5 | 26.65 | 7.83 | 7.63 | 3.45 | -11.25 |
|  |  |  | L | 0.5 | 26.40 | 7.83 | 7.68 | 3.40 | -10.00 |
| 12 | M | 5 | R | 0.50 | 27.18 | 7.72 | 7.39 | 3.60 | -13.50 |
|  |  |  | L | 0.70 | 27.60 | 7.71 | 7.40 | 3.65 | -15.13 |
| 13 | F | 2 | R | 0.40 | 26.36 | 7.61 | 7.35 | 3.52 | -16.00 |
|  |  |  | L | 0.40 | 25.95 | 7.54 | 7.39 | 3.48 | -14.75 |
| 14 | F | 4 | R | 0.20 | 28.23 | 8.39 | 7.92 | 3.46 | -10.63 |
|  |  |  | L | 0.20 | 26.78 | 8.27 | 8.11 | 3.27 | -6.63 |
| 15 | M | 10 | R | 0.10 | 29.52 | 8.05 | 7.69 | 3.75 | -14.00 |
|  |  |  | L | 0.10 | 29.12 | 8.02 | 7.76 | 3.69 | -12.38 |
| 16 | M | 5 | R | 0.10 | 27.75 | 8.31 | 7.83 | 3.44 | -8.63 |
|  |  |  | L | 0.10 | 28.48 | 8.28 | 7.71 | 3.56 | -10.50 |
| 17 | M | 5 | R | 0.10 | 26.17 | 8.21 | 7.98 | 3.23 | -5.88 |
| 18 | F | 5 | R | 0.10 | 28.23 | 7.81 | 7.30 | 3.74 | -13.13 |
|  |  |  | L | 0.10 | 27.47 | 7.79 | 7.23 | 3.66 | -12.25 |
| 19 | M | 10 | R | 0.10 | 27.94 | 7.69 | 7.13 | 3.77 | -13.88 |
|  |  |  | L | 0.10 | 27.34 | 7.66 | 7.05 | 3.72 | -12.88 |
